# “My body’s a 50 year-old but my brain is definitely an 85 year-old”: exploring the experiences of men ageing with HIV-associated neurocognitive challenges

**DOI:** 10.7448/IAS.16.1.18506

**Published:** 2013-07-23

**Authors:** Lisa Hopcroft, Laura Bester, Daniel Clement, Adria Quigley, Manisha Sachdeva, Sean B Rourke, Stephanie A Nixon

**Affiliations:** 1 Department of Physical Therapy, University of Toronto, Toronto, Ontario, Canada; 2 St. Michael’s Hospital, Toronto, Ontario, Canada

**Keywords:** AIDS, disability, rehabilitation, age, Poz-brain, HAND

## Abstract

**Introduction:**

Research investigating HIV, neurocognition and ageing is well developed using neuropsychometric or other quantitative approaches; however, little is known about individuals’ subjective experiences. The purpose of this article is to explore the experiences of men aged 50 and older who self-identify as having HIV-associated neurocognitive challenges. In particular, this study uses the Episodic Disability Framework (EDF) to explore participants’ perceptions regarding: 1) symptoms/impairments, difficulties with day-to-day activities, challenges with social inclusion and uncertainty; 2) ageing as related to their HIV-associated neurocognitive challenges, and 3) the episodic nature of their HIV-associated neurocognitive challenges.

**Methods:**

This qualitative, interpretive study involved in-depth, semi-structured interviews with 12 men aged 50 years and older who self-identified as having HIV-associated neurocognitive challenges. Participants were recruited from a neurobehavioural research unit (NBRU) at a large hospital in Toronto, Canada. Data were analyzed thematically and with reference to the EDF.

**Results:**

Participants’ experiences reflected all concepts within the EDF to some extent. Difficulties with daily activities were diverse but were addressed using similar living strategies. Participants described challenges with work and social relationships resulting from neurocognitive challenges. Participants downplayed the significance of uncertainty in their lives, which they attributed to effective living strategies. Most men reported confusion regarding the link between their neurocognitive challenges and ageing. Others discussed ageing as an asset that helped with coping.

**Conclusions:**

This is the first study to use a disability framework to examine the subjective experiences of men ageing with HIV-associated neurocognitive challenges. Findings reframe the episodic disability experienced by these individuals as being predictably linked to certain triggers. As such, support for managing neurocognitive challenges could focus on triggers that exacerbate the condition in addition to the impairments themselves. The study also describes ageing as not only a source of problems but also as an asset among men growing older with HIV.

## Introduction

Since the introduction of highly active antiretroviral therapy (HAART) in high-income settings in 1996, people with HIV have been living longer lives; quality of life has improved, and morbidity due to HIV infection has declined [1]. For many people who can access and tolerate HAART, HIV has become a chronic disease, which involves complex neurological dimensions [2]. At the same time, challenges related to ageing with HIV have emerged as a clinical, policy and research priority [3–5].

HIV infection can affect the brain directly or indirectly via neoplasms or opportunistic infections [6]. Resulting neurocognitive impairment has been shown to affect up to 52% of people living with HIV [7,8]. There also appears to be an age effect of neurocognitive impairment, with a larger proportion of those affected aged 50 years and over [9]. Criteria for HAND were established by the American Academy of Neurology and include three conditions: Asymptomatic Neurocognitive Impairment (ANI), Mild Neurocognitive Impairment (MNI) and HIV-Associated Dementia (HAD) [10]. ANI affects 30% of people living with HIV [6] and is defined as a deficit at least 1 SD below the mean for age–education-appropriate norms on neuropsychological tests in at least two of the following areas: verbal/language, attention/working memory, abstraction/executive, memory, speed of information processing, sensory–perceptual, and motor skills but no evidence of interference with daily life [10]. MNI affects 20–30% of individuals living with HIV [6] and is similarly defined as a deficit at least 1 SD below the mean for age–education-appropriate norms on neuropsychological tests in at least two areas. However, MNI also includes evidence of at least mild interference on daily functioning [10–13], work performance [14–16], medication adherence [12,17] and quality of life [18]. Finally, HAD, with a prevalence of 2–8% [6] is defined as a deficit at least 2 SD below the mean for age–education-appropriate norms on neuropsychological tests in at least two areas and significant interference with daily functioning [10].

Understanding of HAND has grown tremendously in the recent years through neuropsychometric research and focus on brain function and impairments [2,9,19]; however, little is known about the subjective experience of living with HIV-associated neurocognitive challenges. A key exception is a study by Gallagher *et al*. [20] that explored the experiences of women with HIV-related neurocognitive disorders. This study highlighted the perceived importance of not only the neurocognitive impairments but also the ways in which they manifested in these women’s daily lives and roles [20]. This contribution to the literature by Gallagher *et al*. responds to the chronic underrepresentation of women as participants in HIV research. However, it is also crucial to understand experiences of men with HIV-related neurocognitive impairments, given that men compose of just under half of all people living with HIV worldwide [21]. Furthermore, this inquiry is particularly important for informing HIV responses in resource-rich settings, such as North America and Western Europe, where HIV prevalence among men who have sex with men is understood to play a substantial role in national HIV epidemics [22].

Simultaneous to the growth of interest in HAND has been a surge of research on ageing and HIV in both men and women [4,23], one dimension of which has focused on links to neurocognition [24]. Ageing and HIV appear to have similar effects on neurocognition [2]. Moreover, when tested neuropsychologically, people living with HIV perceive that they “are not ageing as well” as their HIV-negative counterparts [25]. Here too, however, research approaches have privileged quantitative paradigms, which are best suited for clinical research, [2,9,19] over subjective inquiries into the experience of people again with HIV-associated neurocognitive challenges, which can offer insight into novel directions for practice and research.

As such, the purpose of this article is to explore the experiences of men aged 50 and older who self-identify as having HIV-associated neurocognitive challenges. In particular, this study uses the Episodic Disability Framework (EDF) [26,27] to explore participants’ perceptions regarding: 1. symptoms/impairments, difficulties with day-to-day activities, challenges with social inclusion and uncertainty; 2. ageing as related to their HIV-associated neurocognitive challenges; and 3. the episodic nature of their HIV-associated neurocognitive challenges.

## Methods

### Conceptual framework: the “EDF”

Rehabilitation and disability models have been developed to conceptualize the experiences of people with HIV outside a biomedical model. Such approaches have shown great utility for research and policy advocacy due to their attention to and exploration of people’s daily experiences and life challenges. The EDF is a rehabilitation framework developed with people living with HIV to reflect life events associated with transitions between wellness and illness [26,27]. The EDF conceptualizes the multi-dimensional health-related challenges faced by adults living with HIV, including dimensions of episodic disability, factors that describe the context in which disability is experienced, and triggers that may initiate a major or momentous episode of disability.

Specifically, the EDF is composed of four interrelated dimensions of episodic disability: *symptoms/impairments*, *difficulties with day-to-day activities*, *challenges to social inclusion* and *uncertainty* [26]. These four disability dimensions are interlinked such that one dimension is associated with the experience of another. The EDF also includes *extrinsic contextual factors* (such as social support and stigma) and *intrinsic contextual factors* (e.g., include living strategies and personal attributes, such as ageing) that exacerbate or alleviate the experience of disability [26]. The EDF therefore addresses the impairments, activity limitations and participation restrictions of disability, while also acknowledging the episodic, fluctuating and uncertain nature of living with HIV. To date, the EDF has been used to explore experiences of people living with HIV in general [26,27], but has yet to be applied to the experience of HIV-associated neurocognitive challenges, particularly in the context of ageing.

### Study design, sampling and recruitment

This qualitative, interpretive study involved in-depth, semi-structured interviews and chart abstraction to address the research questions. Participants were recruited using convenience sampling [28]. Potential participants were selected from a pool of past research participants associated with the Neurobehavioural Research Unit (NBRU) at St. Michael’s Hospital in Toronto, Canada, who had volunteered to consider future studies. This pool consisted of approximately 750 HIV-positive people who had been referred for neuropsychometric testing by their physicians or self-referred to assess changes in their neurocognitive status. Potential participants were contacted by the NBRU, beginning with those who most recently attended the NBRU. The research team contacted interested individuals by telephone to explain the study. Eligibility criteria included: 50 years or older, HIV-positive, self-identify as having HIV-associated neurocognitive challenges, English-speaking and capacity to consent. Written consent was obtained at the time of the interview. Ethics review committees at St. Michael’s Hospital in Toronto and the University of Toronto approved this study.

### Data collection

An interview guide was designed to explore participants’ perceptions of their HIV-associated neurocognitive challenges according to the EDF dimensions of impairments, activity limitations, participation restrictions and uncertainty. Additional questions explored participants’ perspectives on how ageing and uncertainty may have influenced their experiences. The interview guide was semi-structured and posed primarily open-ended questions (see Table 1).

**Table 1:** Excerpt from interview guide

Can you tell me about how your social relationships have been affected because of your neurocognitive challenges?*
(a) How have your neurocognitive challenges affected your personal relationships? For example, your:
Partners
Family
Friends
(b) How have your neurocognitive challenges affected your professional relationships? For example, your:
Colleagues, co-workers
Employer
Clients
(c) How have your neurocognitive challenges affected your relationships with health care professionals? For example your doctor? Physiotherapist?
(d) Encounters/interactions in the community. For example:
Grocery shopping
At restaurants
On transit
Do you feel that people treat you differently than others who are the same age based on your neurocognitive challenges
Do you think there is a stigma associated with your neurocognitive challenges?
*Probes to ask after each sub-question (i.e. after (a), (b) and (c) are answered)
Could you tell me story of when your relationship was affected?
Do you think the way you interact with others fluctuates? More comfortable some days and not others?
Explain how these relationships may have changed as you have aged.
What do you do to cope with these difficulties? Do you use: Self-talk or positive thinking Relying on family members Support groups Program and policy support e.g., Ontario Disability Support Program, Canada Pension Plan, private insurance plans, and housing and food bank services.

Interviews lasted 30–90 min, and took place in person in a private office at St. Michael’s Hospital between January and April 2011. Each participant received a $25 Canadian honorarium. Interviews were audio-recorded, transcribed verbatim and quality checked to ensure accurate transcription. Demographic and clinical data were collected from participants’ charts at the NBRU to better describe the sample (see Table 2).

**Table 2:** Chart abstraction form

Participant code:	
1. Age	
2. Currently taking HAART medications? Yes/no	
3. Years since HIV diagnosis: ____ years	
4. Comorbidities (list)	
5. Has a HAND diagnosis? Yes/no	
If yes: Severity of HAND	1) asymptomatic neurocognitive impairment (ANI)
	2) HIV-associated mild neurocognitive disorder (MND)
	3) HIV-associated dementia (HAD)
6. Affected areas, e.g. memory, balance, mood (list):	
7. CD4 count: Most recent test date:	
8. Viral load: Most recent test date:	

### Data analysis

Interview data were analyzed thematically, following the open coding technique described by Strauss and Corbin [29]. To begin, each member of the research team read several transcripts to identify patterns of recurring ideas in the data. The team then collaboratively and inductively developed and piloted a coding framework of categories that reflected topics of significance to participants. All transcripts were then coded by two researchers and organized using qualitative data analysis software (NVivo 8.0©). Summaries were generated of the data from each category or “node” in the coding framework. The research team then discussed the major findings for each node to illuminate common themes and patterns of ideas. These findings were then related back to the dimensions of the EDF to consider how or if they applied.

## Results

The 12 participants were male and their ages ranged from 50 to 62 years. All participants had lived in Canada for at least 14 years (see Table 3).

**Table 3:** Summary of participant characteristics

Total number of participants	12
Age in years, mean (range)	55.0 (50–62)
Years with HIV, mean (range)	16.8 (3–29)
Currently on antiretroviral therapy	12
HIV-associated neurocognitive disorders (on neuropsychological testing)	
Normal	4
Asymptomatic Neuropsychological Impairment (ANI)	3
Mild Neurocognitive Disorder (MND)	3
Dementia	2
Depression (according to neuropsychological assessment)	
Minimal complaints	4
Mild complaints	4
Moderate complaints	3
Moderate-to-severe complaints	1
Country of origin	
Canada	8
Others	4
Employment	
Currently working	3
Temporarily off work	2
Early retirement	1
Unemployed	6

Participants’ experiences of living with HIV-associated neurocognitive challenges reflected all dimensions of the EDF to some extent. Findings are organized according to these dimensions: the *impairments* experienced by participants, *challenges with day-to-day activities*, *challenges to social inclusion* resulting from these impairments, *uncertainty*, *ageing* and the *episodic nature* of impairments.

### Impairments

Impairments include symptoms related to body part or function. Nearly all participants reported challenges with attention and/or short-term memory. The men described their lack of attention as difficulty focusing on details for long periods. One man described his memory challenges as follows:

Short-term memory … it’s like, “Oh what was I talking about?” Or “What did we arrange? Did we arrange to meet?” Just gone, I can’t remember. The short-term memory is really getting bad.

Many men described difficulty multi-tasking and, to a lesser extent, being easily distracted. Men also reported problems with decision-making, learning new facts or tasks, problem-solving and word-finding. Some described feeling fatigued and lacking energy. As one participant explained:

I’d compare it [the fatigue] to someone who’s put in a full day of work. Let’s say you’ve gone home, you’ve cooked your meal, cleaned up the house, watched television, played with the kids, [and you are] exhausted. It’s now 11 o’clock, you’ve watched the late news; you’re ready for bed, right? You’re tired. Well that’s how I feel when I wake up in the morning, and it doesn’t get any better.

Other impairments reported by participants included feelings of irritability, apathy, indifference, depression, decreased confidence and challenges with fine motor tasks.

### Difficulties with day-to-day activities

“Activities” refer to typical day-to-day tasks ranging from hygiene to instrumental activities of daily living such as banking. Activities that men reported as difficult were diverse, although participants used similar coping strategies to facilitate task completion. Activity limitations were often due to short-term memory loss (e.g. recalling details of movies, difficulty completing chores). Participants’ living strategies to address these challenges included lists, spreadsheets, calendars and reminders from friends. As one man explained:

I find that I have to write a lot of stuff down now … say I’m going to the grocery store. I’ll get to the grocery store, and I’ll know I need eggs and milk and a bunch of other things, but I don’t remember what they are now. If I don’t have a list, it’s like I’m screwed basically.

Attention impairments were commonly cited impediments to task completion. Men provided examples of some tasks that took longer to finish (such as reading the newspaper), and others that were delayed indefinitely. Some participants reported that their social activities had changed:

If someone said, “Let’s go watch a two-hour movie downtown at the theatre”, I [would] say, “You’re out of your mind. I can’t sit still for longer than 20 minutes. I can’t focus on that.”

Several participants described how fatigue affected their daily activities, resulting in frequent breaks or naps. Many of these participants felt that depression and changes in mood limited their ability to participate in household activities and reduced their interest in activities they previously enjoyed:

I’m depressed about not being able to do something properly or recall a name of someone. I find I’ve dropped a lot of things in my life. You know, a lot of hobbies and interests that I do. Even friends have remarked, “Why don’t you do this often?”

Others limited their activities by choice, as one man stated:

You prioritize whatever in life is important to you. So, you prioritize how you spend your time, [and] do the things that you want to be doing.

### Challenges to social inclusion

Social inclusion refers to an individual’s engagement in social roles, such as being a neighbour, parent, partner or employee. Participants’ narratives addressed three main forms of social inclusion: employment, relationships within the community and personal relationships.

#### Employment

Several participants had retired early because of their neurocognitive challenges. Commonly described barriers to continued employment were decreased ability to focus on tasks, difficulty multi-tasking, forgetting tasks and taking longer to problem-solve. Others explained that they did not have the energy for a job:

By 2 to 3 in the afternoon I’m pretty well pooped. I’m tired and I can’t retain information, [or] make good decisions.

Those still working were concerned about their ability to maintain satisfactory job performance. One man with his own business described:

A client who I told about the neurocognitive challenge, he said that he thought all along that it was the drugs that I was taking. So what I mean by that is, he’s noticed … I told him I have brain damage, which is what I was told.

Men still working had mixed feelings about retirement. Some participants who do not work because of their neurocognitive challenges expressed feelings of guilt about not being a financial provider or a member of the workforce, resulting in feelings of uselessness. Volunteering was described as a way of being productive, however participants emphasized the importance of finding an organization that is flexible with scheduling and accommodating to neurocognitive challenges.

Several of the participants had considered returning to school, but expressed concern about their ability to focus on course work. Some who were taking courses felt their performance was affected: “Those [in depth subjects] put me behind the [rest of the] class because I sit there and I just don’t understand.”

#### Relationships within the community

Several participants acknowledged stigma in the community related to their HIV status and neurocognitive challenges, but also noted coping strategies. One man explained:

I don’t let stigma get to me. There is stigma there of course. There’s stigma in everything, but … just don’t let it get to you.

All of the participants reported generally good relationships with their health care professionals. However, one participant expressed his frustration:

I have to remember what I’m seeing each doctor for because the cancer doctor doesn’t want to hear about HIV, and the HIV guy doesn’t want to hear about the cancer. Well, actually Dr. [for neurocognitive challenges] is good at that in the sense that he kind of says this is all a product of what you’re going through, a combination of everything. But the other ones don’t seem to recognize that.

#### Personal relationships

In terms of social networking, many participants described how they had pruned their circle of friends. Rationales ranged from being overwhelmed with their own lives to simply maturing and “knowing what they want in life now.” Some actively distanced themselves from friends: “As people have said to me, ‘How much do they matter in your life, do they have significance in your life? If not, put them to the side’.”

Others attributed seeing friends less frequently to changing social expectations, their friends having less energy to socialize and a loss of networking that comes with ageing and being out of the workforce. Despite these challenges, those who had support from friends and family deemed it as important to their lives. Many men described cherished relationships with siblings, partners and selected friends, all of whom “accepted them for who they are.” One man discussed how his entire social group was experiencing similar issues:

A lot of my friends are also HIV-positive and about my age, and they’re suffering the same thing. A lot of them have the “CRS”’, which we call “Can’t Remember Shit”. So we’re all in the same boat. It’s almost a joke when we’re all out together.

### Uncertainty

Participants described uncertainty about whether ageing, HIV disease, antiretroviral use or a combination of factors was the cause of their neurocognitive challenges. One participant explained:

I have no idea right now. All I have is questions [about the cause of my neurocognitive challenges]. I don’t know … I don’t want to jump to conclusions, but I’m looking at it in terms that I don’t really know a lot about it. Could it be age? Could it be dementia that’s HIV-related?

Men also expressed feelings of uncertainty about the future, often linked with fears about worsening memory and attention. Men described uncomfortable uncertainty about continuing to work, finances, planning for retirement, being on long-term disability and future loss of independence. Despite acknowledging these stresses, most participants explained that they chose not to fixate on these feelings:

… there’s always uncertainties with something like this because you don’t know what the future holds …. I might be uncertain but I don’t see the point of banging myself around. It doesn’t serve any purpose. It doesn’t do any good. It’s like trying to control something you have no control over. It’s not going to help. So I don’t go on that track.

Conversely, others did not feel uncertain about the future or their neurocognitive status. One participant explained that his memory was still sharp, but that he would feel more uncertainty if it worsened. Another noted:

I don’t worry about those things … I think I’m fortunate. I have a good outlook, my health is very good, I have a good job, I’m a lucky guy. I don’t feel uncertain about the future.

### Ageing

Participants had varying perspectives on the significance of ageing in relation to their experience of neurocognitive changes. Most men described their neurocognitive challenges as worsening over time, but noted that this was also the case for most ageing adults regardless of HIV status. Some men also reported that their uncertainty about the future and their cognitive state has increased over time, but that uncertainty is a natural part of ageing.

When asked to compare themselves to their contemporaries, most commented that they should not have the neurocognitive impairments that they do, considering their age. As one participant reported: “My body’s a 50 year-old but my brain is definitely an 85 year-old.” However, one man thought that he was on par with his siblings and others his age.

Contrary to viewing ageing as problematic, several participants viewed ageing as an asset. While explaining how fatigue affected his ability to work, one man noted:

There are lot of days where I can just run circles around [my younger co-workers] because I have the years of experience and professionalism, and all that stuff where they don’t have that yet.

Another participant explained: “I’m kind of enjoying the aging process – not the memory problems, but getting older you become more relaxed and things just don’t aggravate you as much.”

### Episodic nature of impairments

When asked whether their neurocognitive challenges tended to fluctuate day-to-day, the participants’ responses were mixed. Some felt that their neurocognitive challenges, mood and ability to interact with others could change frequently:

It would change day to day … Some days, I think I can remember the names better. And some days, it’s just like – I can’t remember this.

Others reported their neurocognitive challenges as steady or consistently worsening over time, as exemplified by this participant:

Basically every day, just about this time of the afternoon, 3–4, I start to feel absolutely exhausted, to the point where I can’t even write a phrase.

Some participants identified triggers that would cause their neurocognitive challenges to fluctuate. For example, men reported their challenges worsened when they were feeling overwhelmed or anxious. One participant explained, “[On] your better days it’s easier to be around people, and days when you’re really tired and swamped, it’s not.” Some participants also felt that feelings of depression influenced the severity of their neurocognitive challenges, and vice versa. One man described his challenges with fine motor movements:

Typing has been different again since last summer. I just went into quite a deep depression, and it just slowed everything down.

## Discussion

### Using the EDF to explore neurocognitive challenges

This is the first qualitative study to examine the subjective experiences of men ageing with HIV-associated neurocognitive challenges through the lens of a disability framework. This research approach privileges the perspectives of people affected by neurocognitive disease regarding its impact on their lives, which complements the existing body of research grounded primarily in clinical research. Participants described many neurocognitive challenges consistent with the biomedical literature, with memory and attention being the most commonly impaired [9]. Beyond the impairment level, however, the use of the EDF highlighted how impairments were linked to issues of social inclusion, such as employment and personal relationships. In addition, the EDF focused attention on the living strategies that these men employed to manage their neurocognitive impairments and minimize the impact of the challenges in their daily lives.

The EDF was developed with people living with HIV to describe their episodic fluctuations of disability, which O’Brien *et al*. characterize as “unpredictable periods of wellness and illness” [27]. However, it appears that participants in this study did not find their neurocognitive challenges to fluctuate in the same way as the HIV disease itself. Rather than being unpredictable, these men largely found their HIV-associated neurocognitive impairments to be predictable and linked to identifiable triggers, such as increased fatigue or anxiety. This finding is relevant to practice, as HIV care providers may wish to assist clients with managing the triggers that exacerbate the condition in addition to the impairments themselves.

Consistent with O’Brien [26], participants recognized feelings of uncertainty regarding their future, but described *living strategies* that they employed to manage these feelings [26]. Uncertainty associated with episodic disease has largely been characterized as having a negative influence on people’s experiences living with HIV [26]; however, by engaging positive living strategies, participants described the uncertainty associated with their episodic disease as manageable. These findings emphasize the importance of living strategies in O’Brien’s disablement model, particularly in terms of neutralizing the negative influence of uncertainty. Indeed, the participants were able to identify living (or compensatory) strategies that they use to deal with most of their challenges. The creativity and resilience demonstrated by these men is a reminder for health and social service providers that pragmatic strategies for addressing challenges can be often developed by or with affected communities.

The EDF also enabled discovery of difficulties with social inclusion perceived by the men in this study. This finding reinforces the work of Gallagher *et al*. [20] who identified the importance of participant restrictions by using the World Health Organization’s International Classification of Function, Disability and Health to explore the experiences of women living with HIV-associated neurocognitive challenges [20]. In particular, women in the study by Gallagher *et al*. emphasized issues related to parenting, which was not evident in our data with men [20]. However, both studies highlighted social inclusion challenges related to maintaining employment in the face of fluctuating or deteriorating neurocognition. These difficulties highlight the importance of advocacy efforts to promote more flexible workplace policies and supportive employment environments for people living with HIV and other types of episodic disabilities [30].

### Ageing with HIV-associated neurocognitive challenges

Participants offered insight into the relationship of ageing and their experiences of living with HIV-associated neurocognitive challenges. Despite interest in the literature regarding the precise roles of ageing with respect to neurocognitive pathology [31,32], most of the men in this study were relatively disinterested in the etiology of their problems and focused more on management strategies. These results call for future research on adaptive living strategies and other forms of intervention to ameliorate the challenges of living with HIV-associated neurocognitive disorders.

Ageing is frequently understood as having a negative effect on neurocognition [2,24]. However, several participants viewed ageing as an asset or positive resource, a concept also raised by Emlet *et al*. [33]. For instance, participants described the increased maturity and wisdom achieved as a result of ageing that has enabled them to cope more effectively with their feelings of uncertainty. While certain impairments may worsen with age, the life experience that is gained with age may allow for more effective management strategies. Future practice and research should consider ageing not only as a negative influence in the lives of people living with HIV, but also as a potential asset to be harnessed as a source of strength.

### Limitations

When interpreting the results, it is important to note that most of our study participants were male and born in Canada, a high-income country with a comprehensive social welfare system, including universal health care. Participants were recruited from an urban centre known to have a large HIV community. Furthermore, these participants were recruited from a database of people living with HIV receiving psychological care. As such, we caution against generalizing from the findings of this convenience sample, as their experiences may differ from those who are not engaged with the formal health care system. Future research should explore the experiences of other populations living with HIV-associated neurocognitive challenges who may not enjoy the social structural advantages of this sample.

## Conclusions

The EDF provides a useful lens through which to view HIV-associated neurocognitive challenges because of its ability to link issues of social inclusion to impairments. Furthermore, this framework focuses attention on the living strategies employed by individuals to attend to their challenges, thus highlighting the creativity and resilience of people living with HIV. This study is also novel because of its engagement of the subjective experiences of men living with HIV-associated neurocognitive challenges, which offers an important complement to the literature on HAND that has largely drawn upon quantitative, positive paradigms to date. The findings have implications for HIV health professionals and advocates by highlighting the role of a disability-oriented approach in managing impairments, difficulties with day-to-day activities and challenges to social inclusion.

## References

[1] Gray F, Chretien F, Vallat-Decouvelaere AV, Scaravilli F (2003). The changing pattern of HIV neuropathology in the HAART era. J Neuropathol Exp Neurol.

[2] Ikezu T (2009). The aging of human-immunodeficiency-virus-associated neurocognitive disorders. J NeuroImmune Pharmacol.

[3] Larussa D, Lorenzini P, Cingolani A, Bossolasco S, Grisetti S, Bongiovanni M (2006). Highly active antiretroviral therapy reduces the age-associated risk of dementia in a cohort of older HIV-1-infected patients. AIDS Res Hum Retroviruses.

[4] Luther VP, Wilkin AM (2007). HIV infection in older adults. Clin Geriatr Med.

[5] Stoff DM (2004). Mental health research in HIV/AIDS and aging: problems and prospects. AIDS.

[6] McArthur JC, Steiner J, Sactor N, Nath A (2010). Human immunodeficiency virus-associated neurocognitive disorders: mind the gap. Ann Neurol.

[7] Rourke SB, Carvalhal A, Zipursky AR, Bekele T, McCombe J, Rachlis A (2012). The Canadian “CHARTER” report: prevalence and determinants of HIV-associated neuropsychological impairment in the OHTN Cohort Study.

[8] Heaton RK, Clifford DB, Franklin DR, Woods SP, Ake C, Vaida F (2010). HIV-associated neurocognitive disorders persist in the era of potent antiretroviral therapy: CHARTER Study. Neurology.

[9] Becker JT, Lopez OL, Dew MA, Aizenstein HJ (2004). Prevalence of cognitive disorders differs as a function of age in HIV virus infection. AIDS.

[10] Antinori A, Arendt G, Becker JT, Brew BJ, Byrd DA, Cherner M (2007). Updated research nosology for HIV-associated neurocognitive disorders. Neurology.

[11] Woods SP, Iudicello JE, Moran LM, Carey CL, Dawson MS, Grant I (2008). HIV-associated prospective memory impairment increases risk of dependence in everyday functioning. Neuropsychology.

[12] Heaton RK, Marcotte TD, Mindt MR, Sadek J, Moore DJ, Bentley H (2004). The impact of HIV-associated neuropsychological impairment on everyday functioning. J Int Neuropsychol Soc.

[13] Morgan EE, Woods SP, Weber E, Dawson MS, Carey CL, Moran LM (2009). HIV-associated episodic memory impairment: evidence of a possible differential deficit in source memory for complex visual stimuli. J Neuropsychol Clin Neurosci.

[14] Heaton RK, Velin RA, McCutchan JA, Gulevich SJ, Atkinson JH, Wallace MR (1994). Neuropsychological impairment in human immunodeficiency virus-infection: implications for employment. HNRC Group. HIV Neurobehavioral Research Center. Psychosom Med.

[15] Albert SM, Marder K, Dooneief G, Bell K, Sano M, Todak G (1995). Neuropsychologic impairment in early HIV infection: a risk factor for work disability. Arch Neurol-Chicago.

[16] Heaton R, Marcotte T, White D, Meredith K, Taylor M, Kaplan R (1996). Nature and vocational significance of neuropsychological impairment associated with HIV infection. Clin Neuropsychol.

[17] Hinkin CH, Castellon SA, Durvasula RS, Hardy DJ, Lam MN, Mason KI (2002). Medication adherence among HIV+ adults: effects of cognitive dysfunction and regimen complexity. Neurology.

[18] Trepanier L, Rourke SB, Bayoumi A, Halman M, Krzyanowski S, Power C (2005). The impact on neuropsychological impairment and depression on health-related quality of life in HIV-infection. J Clin Exp Neuropsychol.

[19] Kim S, Miles T, Huber R, Felt MD (2008). Executive cognitive function of older people with HIV/AIDS. J Hum Behav Soc Environ.

[20] Gallagher S, Biro S, Creamer E, Della Rossa E, Collins E, Rourke S (2013). “It’s a hidden issue”: exploring the experience of women with HIV-associated neurocognitive challenges using a disability framework. Disabil Rehabil.

[21] UNAIDS World AIDS day report, 2012 [Internet]. http://www.unaids.org/en/media/unaids/contentassets/documents/epidemiology/2012/gr2012/JC2434_WorldAIDSday_results_en.pdf.

[22] UNAIDS Regional fact sheet 2012: North American, Western and Central Europe [Internet]. http://www.unaids.org/en/media/unaids/contentassets/documents/epidemiology/2012/gr2012/2012_FS_regional_nawce_en.pdf.

[23] Sankar A, Nevedal A, Neufeld S, Berry R, Luborsky M (2011). What do we know about older adults and HIV? A review of social and behavioural literature. AIDS Care.

[24] Hardy DJ, Vance DE (2009). The neuropsychology of HIV/AIDS in older adults. Neuropsychol Rev.

[25] 
Vance DE, Burrage JWJ (2005). Cognitive complaints in adults aging with HIV: a pilot study. Phys Occup Ther Geriatr.

[26] O’Brien K, Bayoumi A, Strike C, Young N, Davis A (2008). Exploring disability from the perspective of adults living with HIV/AIDS: development of a conceptual framework. Health Qual Life Outcome.

[27] O’Brien K, Davis A, Strike C, Young NL, Bayoumi AM (2009). Putting episodic disability into context: a qualitative study exploring factors that influence disability experienced by adults living with HIV/AIDS. JIAS.

[28] Marshal MN (1996). Sampling for qualitative research. Fam Pract.

[29] Strauss A, Corbin J (1998). Open coding. Basics of qualitative research.

[30] Canadian Working Group on HIV and Rehabilitation (CWGHR) http://www.hivandrehab.ca/EN/episodic_disabilities/.

[31] Brew BJ, Crowe SM, Landay A, Lucette AL, Guillemin G (2009). Neurodegeneration and ageing in the HAART era. J Neuroimmune Pharmacol.

[32] Mothobi NZ, Brew BJ (2012). Neurocognitive dysfunction in the highly active antiretroviral therapy era. Curr Opin Infect Dis.

[33] Emlet CA, Tozay S, Raveis VH (2010). I’m not going to die from the AIDS”: resilience in aging with HIV disease. Gerontologist.

